# Gingival Traumatic Ulcerative Granuloma With Stromal Eosinophilia (TUGSE) Mimicking Carcinoma

**DOI:** 10.7759/cureus.98006

**Published:** 2025-11-28

**Authors:** Sanjum Aasma, Shirish K Kujur, Vineeta Gupta, Shashank Agrawal, Jaishree Borkar, Neha Sahu

**Affiliations:** 1 Department of Periodontics, Government Dental College, Raipur, Raipur, IND

**Keywords:** case report, histopathology, mimicking malignancy, oral ulcer, tongue lesion, traumatic ulcerative granuloma with stromal eosinophilia (tugse)

## Abstract

Traumatic ulcerative granuloma with stromal eosinophilia (TUGSE) is an uncommon benign lesion of the oral mucosa that clinically resembles malignancy, often creating diagnostic concern. We report the case of a 46-year-old woman presenting with a persistent gingival growth in the posterior mandible. Clinical examination revealed a firm erythematous mass with a necrotic white plaque. Histopathology and immunohistochemistry confirmed TUGSE, and surgical excision led to complete healing without recurrence. This case highlights the importance of including TUGSE in the differential diagnosis of chronic oral ulcers and emphasizes early biopsy to avoid misdiagnosis, unnecessary aggressive treatment, and patient distress.

## Introduction

Traumatic ulcerative granuloma with stromal eosinophilia (TUGSE) is a rare, benign, but persistent oral mucosal lesion that often mimics malignancy and creates a diagnostic dilemma [1]. It usually presents as a solitary, indurated ulcer with rolled margins and a necrotic base, most commonly affecting the tongue and buccal mucosa due to repeated trauma. Although self-limiting, its alarming appearance frequently leads to concern for squamous cell carcinoma, warranting histopathological confirmation.

TUGSE represents only 0.1% to 0.2% of oral mucosal biopsies, and gingival involvement is exceptionally uncommon, with no precise prevalence established, although several reports describe it as a rare entity occurring predominantly in adults. A slight female predilection has been noted in published case series. Clinically, TUGSE is significant because its ulcerated and indurated presentation may mimic malignant conditions such as squamous cell carcinoma or CD30⁺ lymphoproliferative disorders, necessitating histopathological confirmation for accurate diagnosis.

Histologically, it is characterized by a dense eosinophil-rich inflammatory infiltrate extending into deeper connective tissue, sometimes accompanied by pseudoepitheliomatous hyperplasia that closely resembles carcinoma [2-6]. This report highlights a rare case of gingival TUGSE, underlining the importance of early recognition and biopsy to avoid misdiagnosis and unnecessary aggressive treatment.

## Case presentation

A 46-year-old woman presented with a one-month history of a gradually enlarging gingival growth in the lower right posterior region. She had initially noticed a small lesion that progressively increased in size. Her medical history was non-contributory, and she reported no systemic illnesses or long-term medications. She had been taking ketorolac DT intermittently for pain relief during the symptomatic period.

**Figure 1:**
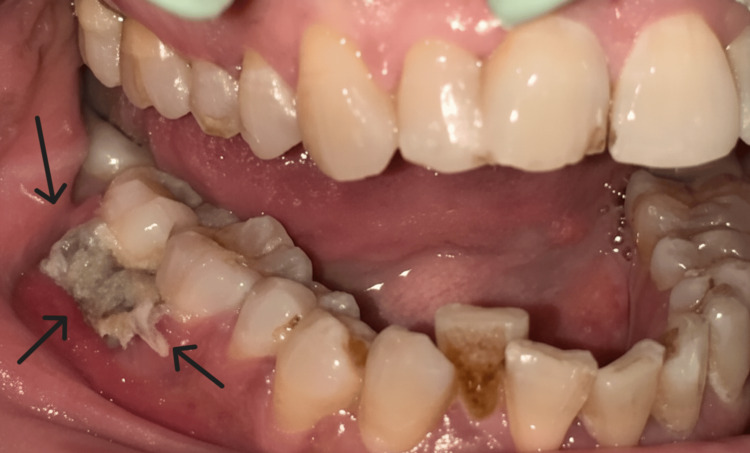
Clinical intraoral photograph A firm, erythematous gingival mass involving the papillary and marginal gingiva of teeth 46 and 47. The lesion displays a non-scrapable necrotic surface plaque and bleeding tendency, features that clinically raise concern for malignancy and support the need for biopsy.

Intraoral examination revealed a firm, erythematous gingival mass involving the papillary and marginal gingiva adjacent to teeth 46 and 47. The lesion measured approximately 1.0 × 1.8 × 0.6 cm, exhibited well-defined borders, and was covered by a non-scrapable necrotic white plaque. The lesion bled on manipulation (Figure 1). The patient reported localized pain persisting for nearly one month. The involved teeth were non-tender on percussion, and periodontal probing revealed a 5 mm pocket in the affected area.

**Figure 2:**
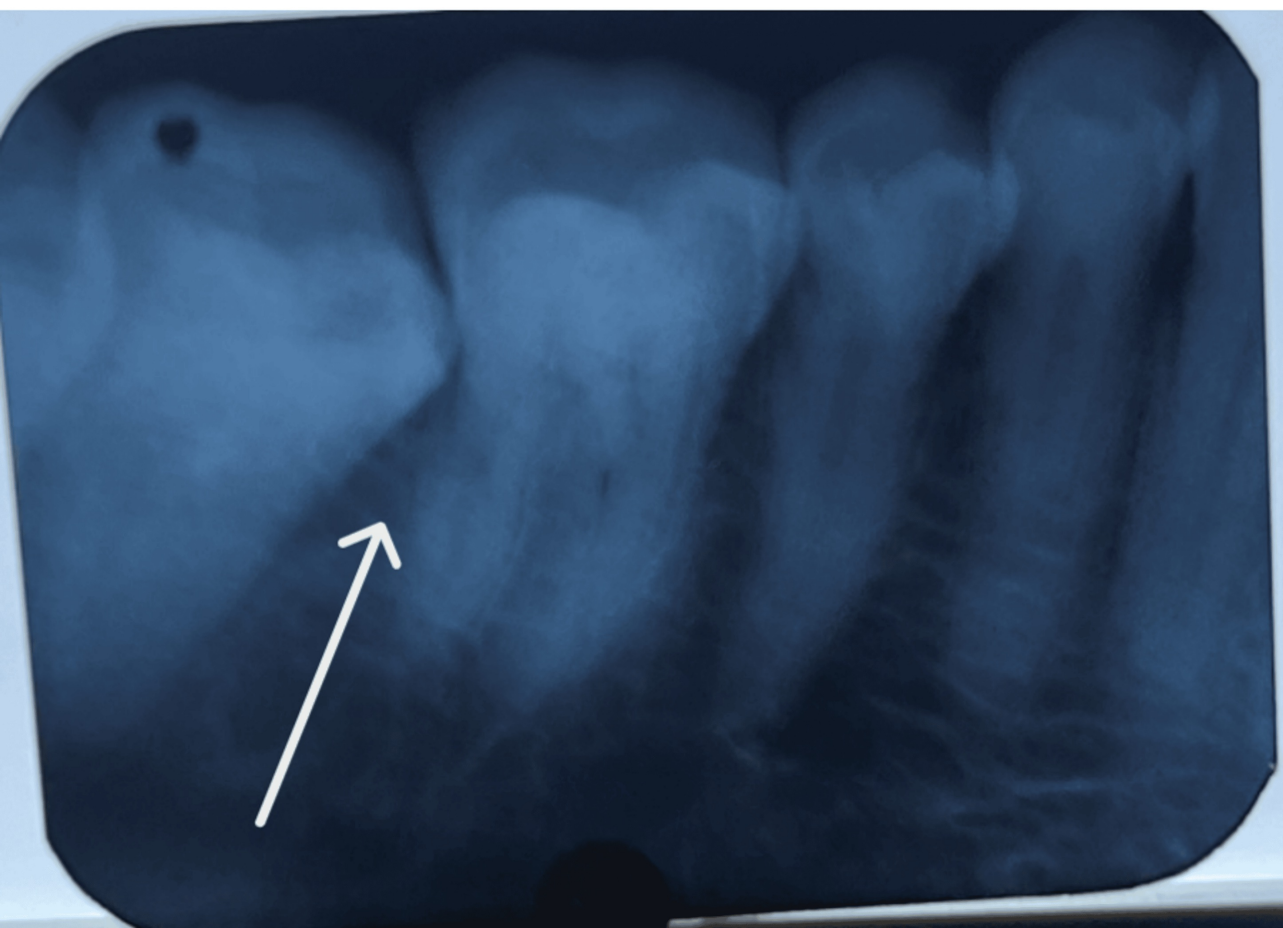
Periapical radiograph localized bone loss extending to the cervical third of the mesial root of tooth 47. This radiographic involvement helps differentiate reactive lesions from potentially aggressive neoplastic processes.

Radiographic examination revealed bone loss extending to the cervical third of the mesial root of tooth 47 (Figure 2). Following scaling and root planing, an excisional biopsy was performed. Histopathological evaluation demonstrated polypoid tissue covered by extensively ulcerated stratified squamous epithelium. The underlying connective tissue exhibited a dense mixed inflammatory infiltrate comprising histiocytes, numerous eosinophils forming microabscesses, and small lymphocytes, accompanied by prominent vascular proliferation and focal areas of necrosis.

**Figure 3:**
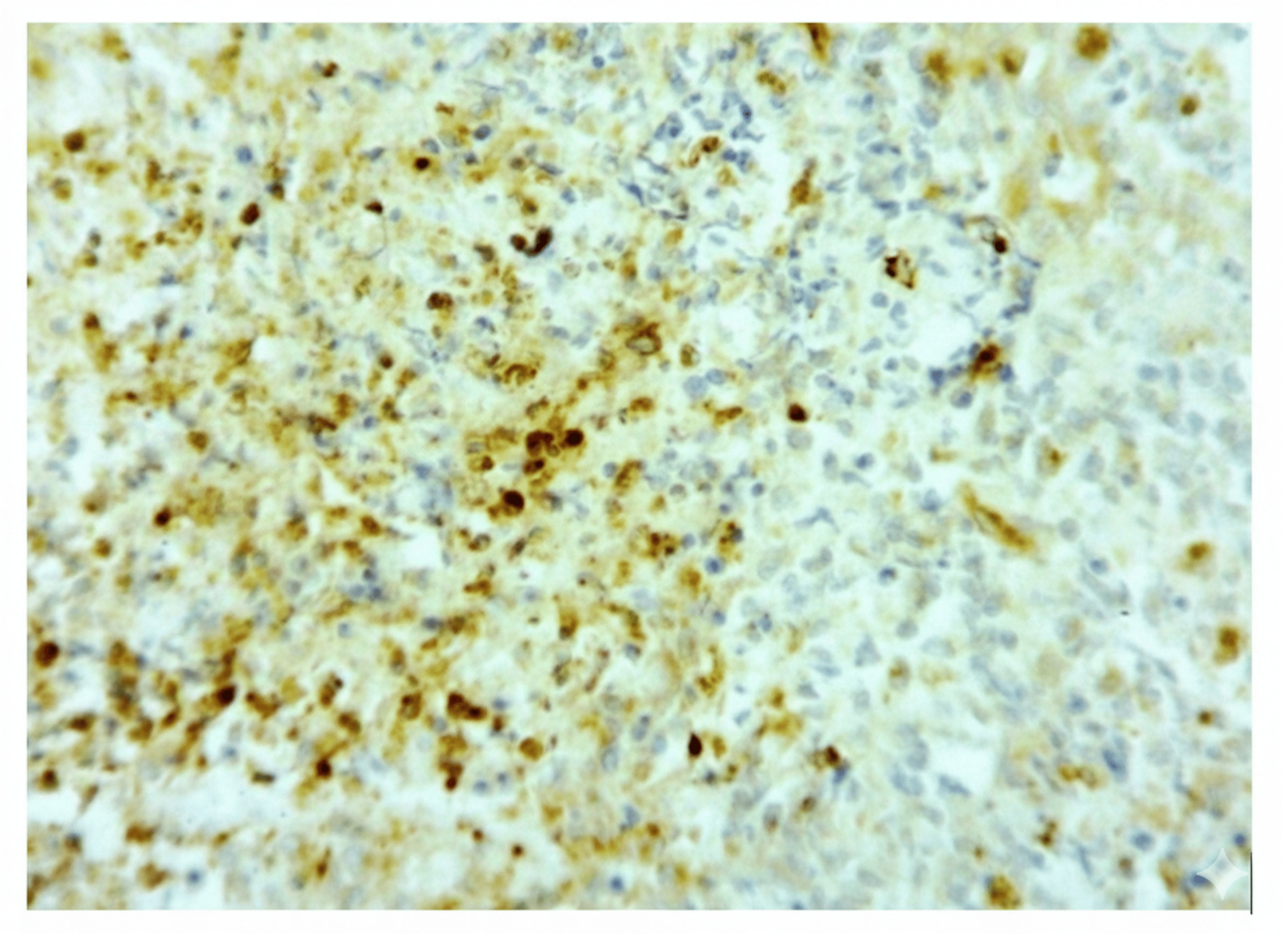
Histopathological view (40x) Microscopic image (H&E stain, ×100) showing polypoid tissue with ulcerated squamous epithelium and underlying mixed inflammatory infiltrate. Numerous eosinophils forming microabscesses, lymphocytes, and vascular proliferation with areas of necrosis are evident.

**Figure 4:**
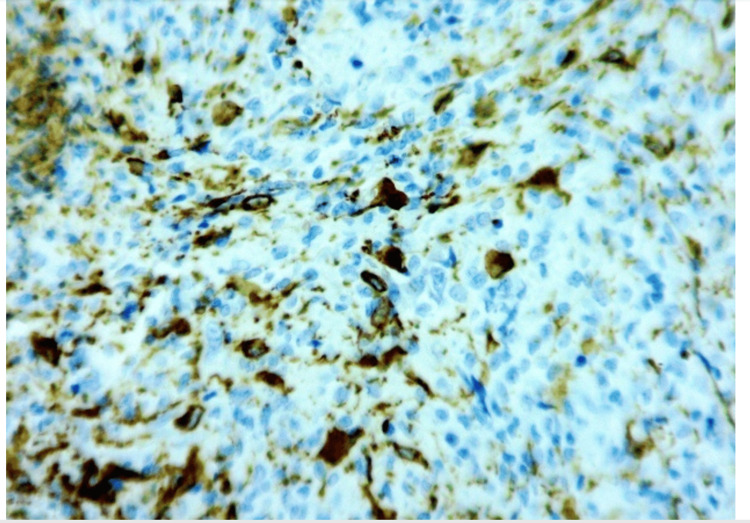
Immunohistochemistry of lesional tissue (40x) Immunohistochemistry of lesional tissue demonstrating positivity for CD163, CD68, and FXIIIa, with focal S100 reactivity (×200). Scattered cells express CD1a and CD10, while staining is negative for langerin, CD21, CD23, CD34, CD35, ALK, and SMA.

Immunohistochemical analysis showed diffuse positivity for CD163, CD68, and FXIIIa, with focal reactivity for S100 (Figure 3). Scattered cells demonstrated expression of CD1a and CD10. The lesion was negative for langerin, CD21, CD23, CD34, CD35, ALK, and SMA (Figure 4).

The histological and immunophenotypic profile confirmed TUGSE. Langerhans cell histiocytosis was excluded due to the absence of langerin and strong CD1a expression, while angiolymphoid hyperplasia with eosinophilia was ruled out by the lack of lymphoid follicles. Following scaling and root planing, the lesion was completely excised. Healing was uneventful, with complete resolution and no recurrence during follow-up.

## Discussion

TUGSE is a rare, reactive oral lesion that frequently mimics malignancy. It typically presents as a solitary indurated ulcer or polypoid growth, most often on the tongue or buccal mucosa, while gingival involvement is unusual. Because clinical features alone cannot reliably exclude squamous cell carcinoma, histopathological confirmation is essential. In the present case, the initial impression on routine H&E staining was suspicious for malignancy, highlighting the diagnostic dilemma.

Histologically, TUGSE demonstrates a polymorphic inflammatory infiltrate composed of histiocytes, lymphocytes, and abundant eosinophils extending deep into connective tissue, sometimes involving muscle or salivary glands. Immunohistochemistry further supports its reactive nature, with positivity for CD68 and CD163 indicating macrophage activity, while the absence of langerin and strong CD1a expression helped rule out Langerhans cell histiocytosis [2,3,6]. Similar findings have been emphasized in recent reports, where eosinophils and activated T-lymphocytes are considered central to tissue damage and repair.

The etiopathogenesis remains incompletely understood, though trauma is strongly implicated. Eosinophils likely contribute through the release of cytotoxic proteins and cytokines that perpetuate inflammation. Other etiologies with histological findings are presented in Table 1, such as CD30-positive lymphoproliferative disorders or certain infections like primary syphilis or Epstein-Barr virus-related mucocutaneous ulcers [7-10]. Although the precise pathogenesis remains uncertain, local trauma is strongly implicated as a major contributing factor. This traumatic origin was first proposed by Fede in 1890, following a comprehensive review of similar lesions in humans [11]. In the present case, excision achieved complete resolution with no recurrence.

**Table 1:** Differential diagnosis of TUGSE TUGSE: traumatic ulcerative granuloma with stromal eosinophilia; SCC: squamous cell carcinoma; CD30+ LPD: CD30-positive lymphoproliferative disorder; EBV: Epstein–Barr virus; IHC: immunohistochemistry; HRS: Hodgkin/Reed–Sternberg cells; TPPA: *Treponema pallidum* particle agglutination; FTA-ABS: fluorescent treponemal antibody absorption test; TNF-α: tumor necrosis factor alpha

Parameter	TUGSE	SCC	CD30+ LPD	Lues (Syphilis)	EBV Mucocutaneous Ulcer	Aphthous Stomatitis
Location in the Oral Cavity	Tongue, buccal & vestibular mucosa, palate, retromolar area, gingiva, floor of mouth [1–6]	Floor of mouth, lateral/tip of tongue, lower lip, retromolar area [10]	Rarely oral; often recurrent [8]	Lips, tongue, pharynx [11]	Oropharyngeal mucosa [9]	Labial/buccal mucosa, soft palate, ventral tongue; severe cases may affect keratinized mucosa [12,13]
Etiopathogenesis	Unknown; often linked to trauma [1,2,4,11]	Chronic tobacco/alcohol use, poor oral hygiene [10]	Seen in immunocompromised patients [8]	Sexually transmitted or congenital infection [11]	Linked with immunosuppression [9]	Stress, trauma, hormones, allergies (foods, toothpaste) [12,13]
Peak Age	Bimodal: infants & 50–70 yrs [1,2,5]	>50 yrs; males > females [10]	Elderly (>75 yrs) [8]	30–40 yrs; males > females [11]	Mostly elderly (>75 yrs); can occur at any age [9]	Any age; more common later in life [12,13]
Clinical Features	Indurated ulcer with yellow fibrinous base [1–3,6]	Endophytic nodular/shallow ulcer with raised, infiltrative margin; non-removable reddish/whitish patches [10]	Indolent nodules or ulcers may have skin manifestations [8]	Painless indurated superficial ulcer (chancre) 2–3 weeks post exposure [11]	Well-demarcated, indolent ulcer [9]	Painful shallow round/oval ulcers with erythematous border and grayish-white pseudomembrane [12,13]
Histopathology	Granulomatous tissue with eosinophils, histiocytes; extends into muscle/salivary glands [1–6,13]	Dysplastic epithelium with keratinization (horn pearls); plasma cell-rich inflammation [10]	Atypical lymphoid infiltrate with eosinophils in deep tissue [8]	Plasmacytosis, vague granulomas; spirochetes visible with Warthin–Starry stain [11]	Atypical large B-cells (HRS-like), polymorphous infiltrate, sharply demarcated [9]	Non-specific inflammation with T-cells; high TNF-α [12,13]
IHC	T-lymphocytes; occasional CD30+; CD68+, CD163+, FXIIIa+, focal S100+ [2,6,13]	CK5/6+, CK19+, p63+, p40+ [10]	MUM1p+, MYC+, CD30+ T-cells; loss of normal markers [8]	Treponema pallidum detection via TPPA and FTA-ABS tests [11]	EBER+, CD30+ [9]	Non-specific [12,13]

Awareness of this benign entity is crucial to prevent unnecessary aggressive surgery and to reduce the psychological burden associated with a suspected malignancy. 

Persistent gingival ulcers can clinically mimic squamous cell carcinoma, making early biopsy essential. TUGSE is characterized by eosinophil-rich inflammation with immunohistochemical features supporting a reactive process. Careful histopathology and immunoprofiling help rule out malignancy, lymphoproliferative disorders, and infectious causes. Most cases resolve spontaneously or after excision, underscoring the importance of timely recognition to avoid overtreatment.

In TUGSE, management primarily involves eliminating traumatic factors and monitoring for spontaneous resolution. Persistent or symptomatic lesions typically require surgical excision, which remains the most predictable and evidence-supported treatment. Adjunctive periodontal therapy, corticosteroids, or laser excision may be used in select cases. Antibiotics are reserved only for secondary infection. Regular follow-up is essential to ensure complete healing and exclude recurrence. The patient described feeling anxious when she noticed the growth on her gums, fearing that it might be cancer. After the biopsy and treatment, she expressed relief and gratitude for the quick recovery. This case emphasizes the importance of considering TUGSE as a differential diagnosis for chronic oral ulcers. Recognition of this benign but alarming lesion prevents misdiagnosis, unnecessary treatment, and anxiety for both patients and clinicians.

## Conclusions

TUGSE is a rare but clinically important differential for persistent oral ulcers and growths, particularly when they resemble squamous cell carcinoma. Definitive diagnosis requires histopathological and immunohistochemical evaluation. Early recognition ensures appropriate management, prevents overtreatment, and reassures patients of its favorable prognosis. The patient was followed for three months after surgical excision, during which the site exhibited complete mucosal healing with no evidence of recurrence.
